# Paraspinal Extrarenal Wilms Tumor Case Report and Review of Literature

**DOI:** 10.1155/crpe/8820759

**Published:** 2026-05-27

**Authors:** Julianna L. Barbaro, Violet M. Borowicz, Brendan J. Klein, Anthony J. Emanuel

**Affiliations:** ^1^ Virginia Tech Carilion School of Medicine, Virginia Tech, Roanoke, Virginia, USA, vt.edu; ^2^ Translational Biology, Medicine and Health Graduate Program, Fralin Biomedical Research Institute at VTC, Roanoke, Virginia, USA; ^3^ Department of Pediatrics, Section of Pediatric Hematology/Oncology, Carilion Clinic, Roanoke, Virginia, USA, carilionclinic.org; ^4^ Department of Neurosurgery, Carilion Clinic, Roanoke, Virginia, USA, carilionclinic.org; ^5^ Dominion Pathology Associates, Carilion Clinic, Roanoke, Virginia, USA, carilionclinic.org

**Keywords:** extrarenal Wilms tumor case report, lumbosacral mass, triphasic histology

## Abstract

**Introduction:**

Extrarenal Wilms tumors (ERWTs) (i.e., nephroblastoma) are exceptionally rare tumors that have only been reported approximately 100 times in the literature. These tumors necessitate histology (rather than imaging) for proper identification, often resulting in a postoperative diagnosis.

**Case Presentation:**

At 20 weeks of gestation, a female fetus was diagnosed with a subcutaneous lumbosacral mass by prenatal ultrasound (US). Days after birth, the mass was resected and pathologically determined to be an ERWT. Specifically, the excised mass had a triphasic histologic pattern, including blastemal, stromal, and primitive epithelial components. Centrally, the lesion demonstrated cystic and pseudopapillary architectural features, while peripherally, the lesion was more solid with the morphologic appearance of Wilms tumor (WT). This case is unique due to (1) the unusual lumbosacral location, (2) the presence of normal bilateral kidneys on US, and (3) the detection of the mass on in utero imaging studies.

**Literature Review:**

Including our report, ERWT has been reported 16 times in the (para)spinal/vertebral region and range from T9 to the coccyx. Most patients initially receive surgery (gross total resection, if feasible) followed by chemotherapy (vincristine and dactinomycin) and radiation depending on final pathology and additional case considerations.

**Discussion:**

Due to the rarity of ERWTs, there is no standardized treatment. Complete excision with adjuvant chemotherapy (vincristine and dactinomycin) is most often suggested as a best/most appropriate approach to treatment, with the addition of radiotherapy for recurrence and/or metastasis.

**Conclusion:**

This case report and literature review highlights the necessity of considering ERWT as a potential diagnosis when faced with a patient who has a lumbosacral paraspinal/spinal mass, even if other clinicoradiographic features typical of WT are not identified. We also call to light the need for a standardized treatment regimen for ERWT.

## 1. Introduction

Wilms tumor (WT), also known as nephroblastoma, accounts for approximately 95% of renal tumors in pediatric patients and 5% of childhood cancers overall, with a peak incidence at 3 years of age [1–3]. However, cases of extrarenal WT (ERWT), which were first identified in 1961, account for 0.5%–1% of WTs. These are most commonly located in the retroperitoneum and have only been reported approximately 100 times in the literature [1, 3, 4].

Generally, it is believed that cases of ERWT form along the craniocaudal primitive meso‐ and metanephros cells’ migration pathway. ERWTs have been described in the mediastinum, retroperitoneum, inguinal region, pelvis, adrenal glands, bladder, colon, lumbosacral region, paravertebral soft tissues, spinal cord, and both male (prostate, scrotum, and testis) and female (uterus, ovary, and cervix) genitalia [1].

Initial diagnostic techniques for ERWT include ultrasound (US), computerized tomography (CT), and magnetic resonance imaging (MRI). ERWT does not present with specific radiological features, so histologic evaluation is necessary to differentiate ERWT from other primary and/or metastatic tumors [1]. Moreover, ERWT are “unstageable” by conventional criteria, resulting in no standardized treatment and no available long‐term survival data [5].

## 2. Case Presentation

A 0‐day‐old female, born at 37 weeks and 3 days of gestation via a scheduled Cesarean section to a 36‐year‐old G9P6025 mother, presented after delivery for surgical evaluation of a known subcutaneous lumbosacral mass. Maternal complications included advanced maternal age, gestational diabetes, intrauterine growth restriction, and hyperthyroidism (Graves). The mother was syphilis, HIV, Hepatitis B, Hepatitis C, gonorrhea, and chlamydia negative, as well as rubella immune, and GBS unknown.

**FIGURE 1:**
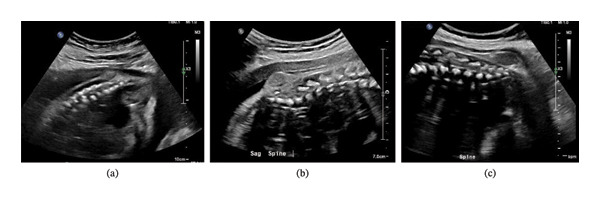
US of the fetus in utero. (a) 27 2/7 weeks GA by LMP (26 1/7 weeks GA by US) demonstrating a 2.17 × 1.72 × 0.8 cm mass on the skin of the sacrum. (b) 30 5/7 weeks GA by LMP (29 2/7 weeks by US) demonstrating a 17 × 15.5 × 8.5 mm sacral mass. (c) 34 5/7 weeks GA by LMP (32 1/7 weeks by US) demonstrating 3.4 × 2.3 × 2.9 cm sacral mass.

**FIGURE 2:**
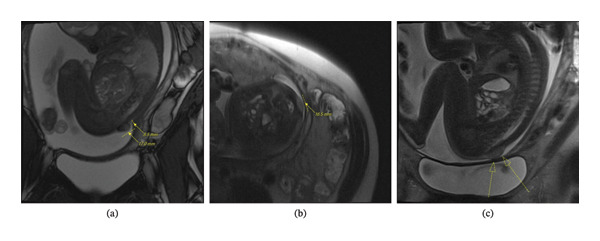
MRI of the fetus in utero. (a) Sagittal. (b). Transverse. (c) Sagittal. MRI findings demonstrated a 17 × 16.5 × 8.5 mm discoid homogenous T2 mildly hyperintense soft tissue mass at the base of the spine.

Prenatally, the patient was found to have a lumbosacral mass by imaging; this was first identified on US at 27 weeks gestational age (GA, based on last menstrual period) (Figure 1a), and it was confirmed by MRI at 29 weeks GA (Figure 2a‐b) and follow‐up US at 30 and 34 weeks GA (Figure 1b‐c). One other notable structural abnormality was an absent septum pellucidum. Overall, the fetal anatomy did not suggest fetal aneuploidy, and the mass was thought to represent a Type 1 sacrococcygeal teratoma.

**FIGURE 3:**
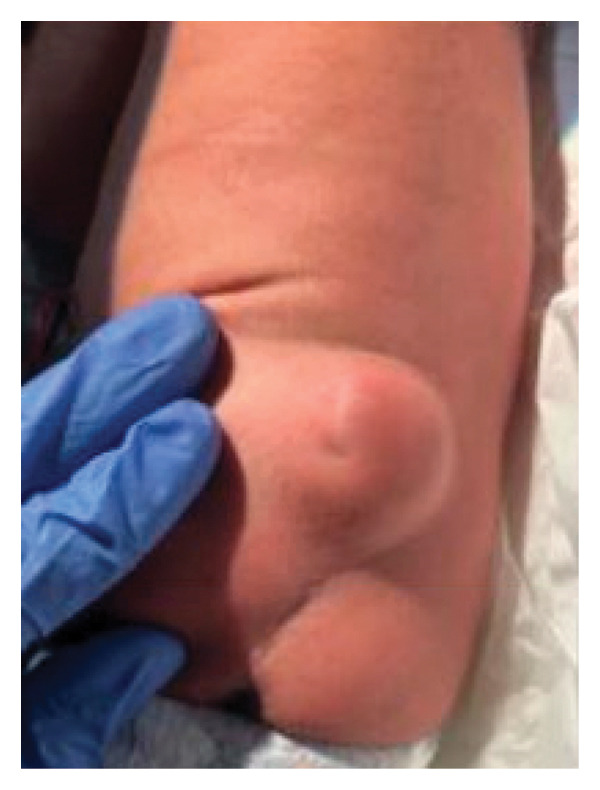
Subcutaneous lumbosacral mass present on the newborn at birth. Image taken within a few days of birth.

At birth, the baby weighed 2170 g (< 1^st^ percentile), with a head circumference of 32 cm (2^nd^ percentile) and a length of 45 cm (1^st^ percentile). The baby had APGAR scores of 8 and 9 at 1 and 5 min, respectively. Physical exam was unremarkable except for a 4 × 4 cm firm lumbosacral mass positioned slightly to the left of midline superior to the coccyx and sacral vertebrae, with normal coccyx on exam. The skin was intact with no open sinus or drainage tracts, and a small dimple was present in the right lateral region (Figure 3). Rectal exam revealed a normal anus with no identifiable internal component of the mass. The baby passed meconium following delivery. The patient was referred to neurosurgery for further evaluation, with an initial differential diagnosis including meningocele and sacrococcygeal teratoma based on the clinical and radiographic findings. No neurological deficits were visible.

**FIGURE 4:**
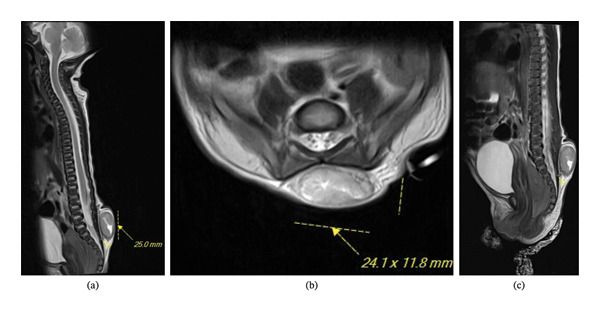
MRI of a 1‐day‐old female. (a) Sagittal T2 image demonstrating a tethered conus medullaris terminating at L3 with an intradural juxtamedullary (subpial) conus lipoma extending dorsal (L2) and caudal (L5) and measuring up to 0.3 cm in axial diameter. (b) Axial image depicting a 2.4 × 1.2 cm axial (2.5 cm craniocaudal) transmidline circumscribed mass between superficial cutaneous tissue and superficial myofascial plane spanning from L4 to S3. (c) Sagittal T2 image demonstrating potential sinus tract extending through dorsal margin of canal at S3 upper aspect.

Chest x‐ray (CXR), abdominal US, and echocardiogram (ECG) were normal. MRI of the patient at 1 day of age revealed multiple abnormalities, including (1) a lumbosacral subcutaneous mass between the skin and superficial myofascial plane with potential extension of a sinus tract through the epidural fascia without intrathecal extension and (2) a L3 dorsal spinal lipoma tethering the spinal cord and a low‐lying conus medullaris (Figure 4a–c).

Neurosurgery elected to remove the lumbosacral mass acutely, with plans to remove the spinal lipoma at 8–12 months of age. Operative findings included gross total resection (GTR) of an approximately 3 cm, tan/yellow, encapsulated cystic mass. Following en bloc removal, the mass was incised intraoperatively, revealing thin yellow fluid, fat, and sebum, with no hair or other features to suggest teratoma. There was an associated posterior midline fibrous tract, with a large penetrating vein, traversing a small opening in fascia with extension to the epidural space. There was no obvious connection with the cerebrospinal fluid (CSF) space or thecal sac. There was no CSF leakage during a Valsalva maneuver, and no other surgical complications were observed.

**FIGURE 5:**
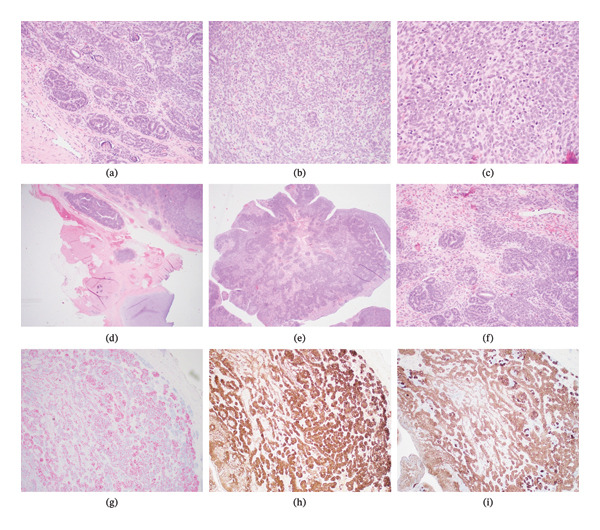
Histology and immunohistochemistry of paraspinal ERWT. (a) (H&E, 200x). Medium power view demonstrating epithelial component of tumor. (b) (H&E, 200x). Medium power view demonstrating stromal component of tumor. (c) (H&E, 400x). High power view demonstrating blastemal tissue. (d) (H&E, 20x). Low power view demonstrating tumor (top) and background paraspinal soft tissue (bottom). (e) (H&E, 40x). Low power view demonstrating triphasic tumor. (f) (H&E, 200x). Medium power view demonstrating triphasic tumor, including blastemal (yellow arrow), stromal (red arrow), and epithelial (black arrow) components. (g) (Ki‐67 immunostain, 40x). Markedly increased proliferative index throughout tumor, estimated at 30%–40% overall. (h) (PAX‐8 immunostain, 40x). Diffuse and strong PAX‐8 expression in tumor (supporting renal origin). (i) (WT‐1 immunostain, 40x). Diffuse and strong WT‐1 expression, particularly in epithelial and blastemal components.

Following resection, the tumor was sent to pathology for evaluation. Histologically, the tumor was found to be a triphasic neoplasm with morphologic features classic for WT. Specifically, this included a cellular proliferation with malignant epithelial, stromal, and blastemal tissue. There was site‐appropriate background fibroconnective, adipose, and cartilaginous tissue, though these structures were not part of the neoplasm. The epithelial structures within the neoplasm included variably well‐formed glomeruli and primitive tubules (Figure 5a). The stromal component of this case primarily included nondescript spindle cells/fibrous tissue with mild to moderate cytologic atypia (Figure 5b). Finally, the blastemal component was characterized by tightly packed spindled to epithelioid cells with high nuclear to cytoplasmic ratios (Figure 5c). All three components exhibited increased mitotic activity, including rare atypical mitoses. While a teratoma was considered, this case lacked other tissue elements other than those noted above (i.e., no evidence of other mature or immature epithelial, mesenchymal, or neural elements). Notably, the WT was without evidence of focal or diffuse anaplasia. The tumor appeared to be narrowly excised in most of the planes of sectioning examined. There was focal extension of tumor cells to an inked and cauterized tissue edge in the main lesion, likely due to the operative need to truncate the base of the tumor in order to follow the fibrous tract and feeding vein deeper through the fascia. An additional tissue fragment overlying the area of tumor on ink was submitted as a second specimen, and this was negative for malignancy, ultimately corresponding with complete excision of the tumor and benign margins.

Although the morphologic appearance of the tumor was classic for WT, a panel of immunohistochemical stains was performed. This ultimately supported the diagnosis of WT (Table 1); in particular, the tumor demonstrated strong and relatively diffuse immunoreactivity for PAX‐8 (Figure 5h), WT‐1 (Figure 5i), and cytokeratin AE1/AE3 (mostly within epithelial component). The negative stains (Table 1) argued against other tumors within the differential diagnosis. Finally, a Ki‐67 (Figure 5g) stain was markedly elevated in the tumor, supporting an increased proliferative index.

**TABLE 1:** Immunohistochemical staining.

Marker	Result
CD45	Negative; highlights background small lymphocytes
CD99	Positive in majority of stromal and blastemal components Patchy weak expression in epithelial component
Desmin	Negative
GFAP	Negative
Keratin AE1/AE3	Positive in epithelial component of tumor
Ki‐67	Elevated proliferative index throughout tumor, estimated at 30%–40% overall
P53	Focal weak expression, compatible with wild‐type pattern
PAX‐8	Diffuse and strong nuclear expression throughout majority of tumor
SOX‐2	Negative
Synaptophysin	Negative
Vimentin	Negative in majority of epithelial component Highlights stromal and blastemal elements of tumor
WT‐1	Diffuse and strong nuclear expression throughout majority of tumor

To assist with further prognostic assessment of the tumor, a DNA methylation array analysis was performed. This analysis failed to detect a methylation class above the reportable threshold, further excluding other tumors in the differential diagnosis; as WT material was not specifically included in the assay for comparison, the negative classification result is not entirely unexpected. This assay was also negative for identifiable large‐scale chromosomal gains or losses. In the context of WT, this is notable for a lack of adverse prognostic markers, including (but not limited to) chromosome 1q gain, chromosome 1p or 16q loss of heterozygosity (LOH), and abnormalities involving MYCN or TP53.

Overall, the pathologic findings supported ERWT with favorable histology and no features of anaplasia. Following a comprehensive evaluation, including multidisciplinary tumor board discussion, the plan was to monitor the patient for kidney abnormalities, such as the development of a renal WT, via abdominal US and CXR, aiming to avoid contrast CT studies given the young age. MRI was also planned at 6 months of age to assess for recurrence of the lumbosacral mass, as well as to monitor the spinal lipoma. Chemotherapy was considered, but given the favorable histology, benign margins, and lack of adverse genetic findings, no adjuvant chemotherapy is currently planned.

At the patient’s 2‐ and 6‐week postoperative visits, the incision was healing well, her legs were moving without evidence of deficit, and there were no fevers, other signs of infection, or apparent bowel or bladder issues. The patient will continue to be evaluated, as above, to assess for tumor recurrence and the need for additional therapy.

## 3. Literature Review

A review of the literature was conducted to identify all spinal and paraspinal ERWT. Including the current case, there are 11 documented paraspinal ERWT and 5 documented spinal ERWT. Most patients were female (14/16) and ≤ 4 years old (14/16), with up to 4 cases being diagnosed before reaching an age of 1 month (two cases listed “newborn,” without further specification) (Table 2).

**TABLE 2:** All documented spinal and paraspinal ERWT.

Report	Age/sex	Spinal vs. paraspinal	Location	Imaging findings	Congenital anomalies	Histology/pathology	Treatment	Outcome
Our case	0 DOF	Paraspinal	L4–S3 subcutaneous mass	MRI: normal kidneys, 2.4 × 1.2 cm subcutaneous mass	Tethered cord, subpial L2–L5 lipoma	Triphasic, prominent tubules and poorly formed glomeruli, spindle stromal cells with a myxoid appearance, mitotic blastemal cells	GTR	Disease free at 6 weeks postop
[6]	22 YOF	Spinal	L4–S1 spinal canal (intramedullary)	MRI: enhancing 6 × 5 × 3 cm intramedullary mass US: kidneys normal	Lipomyelomeningocele	Primitive WT‐1+ blastemal cells, spindle‐shaped stromal cells, glomeruloid/tubular epithelial tissue with no anaplastic/teratomatous tissue	Laminectomy + GTR	Motor improvement; persistent urinary incontinence
[3]	Newborn F	Paraspinal	T12–L5 meningomyelocele sac	MRI: normal kidneys, lower thoracic‐lumbar vertebrae defect, heterogenous T2 hyperintense, T1 hypointense 18 × 30 × 27 mm mass inside CSF‐filled myelomeningocele pouch	Meningomyelocele	Triphasic WT‐1+ blastemal/epithelial tissue. No anaplastic/teratomatous tissue	GTR + CT	No recurrence at 2 months
[1]	4YOM	Spinal	T9–S4 irregular solid spinal canal mass	MRI: normal kidneys, intramedullary thoracolumbar mass with extramedullary growth, C1‐C2 oval shaped mass, medial left temporal bone leptomeningeal tumor metastasis	Spinal dysraphism (spinal bifida)	Triphasic WT‐1+ hyperchromic nuclei in mitotic blastemal cells, renal tubules and primitive glomeruli in epithelial cells, fibrous stromal tissue	Excision impossible, chemotherapy (etoposide [150 mg/m^2^], carboplatin [200 mg/m^2^], cyclophosphamide [450 mg/m^2^], doxorubicin [50 mgm^2^]) + RT (25.5 Gy in 17 fractions) followed by palliative chemotherapy(ifosfamide [2000 mg/m^2^], carboplatin [500 mg/m^2^], etoposide [100 mg/m^2^])	Death 2 years after initial diagnosis
[7]	3 YOF	Spinal	L2–S2 spinal canal	MRI: normal kidneys, L2–S2 mass involving the spinal cord	Dermal sinus, tethered cord	Stage 3 triphasic tumor	STR + chemotherapy + RT	Residual lower‐extremity weakness (4/5)
[8]	Newborn F	Paraspinal	L5 dorsal paraspinal mass	MRI: normal kidneys, 2 × 2 × 1 cm uniformly enhancing T1 isointense, T2 hyperintense mass	Dysraphic lamina + intradural L1‐L2 lipoma	2 × 1.7 × 1 cm encapsulated, cystic, globular mass with blastema, tubules, glomeruli and mesenchyme, no teratomatous tissue	GTR + 18 weeks of chemotherapy (vincristine, dactinomycin)	Disease‐free at 2.5 years postop
[9]	20 DOM	Paraspinal	Lumbosacral L4‐L5 extradural mass	MRI: heterogenous solid mass	Spina bifida + tethered cord + spinal dysraphism	Wilms tumor component within immature teratoma	GTR	Disease free at 9‐month postop
[10]	18 MOF	Paraspinal	L1 paraspinal mass, diastematomyelia	MRI: intradural extramedullary heterogenous, hypointense T1, hyperintense T2, L2–L5 mass	Diastematomyelia + bone spur + tethered cord at L1	Triphasic component, with blastema having hyperchromic vesicular nuclei with frequent mitosis, large amount of tubules, and some primitive glomeruli	GTR + CT	Disease free at 3 months, still on CT
[11]	1 YOM	Paraspinal	L2–L4 paraspinal mass	CT: solid 5 × 4 × 5 cm paraspinal mass	None reported	Tubules and glomeruli in blastemal‐like undifferentiated cells with round‐to‐oval nuclei. No anaplastic features	GTR + chemotherapy + RT	Undergoing chemotherapy + RT
[12]	4 YOF	Paraspinal	T10–coccyx extradural sacral mass	CT/MRI: mixed‐intensity mass from pelvic brim to femoral head with extension through neuroforamina and attachment to gluteal muscles	Spina bifida occulta, no spinal dysraphism	Triphasic, spindle cell mesenchyme, primitive mitotic tubular with hyperchromatic nuclei and glomeruloid epithelium, focal primitive blastema, neither anaplasia nor rosettes nor neuropil nor teratomatous tissue, limited necrosis	No response to chemotherapy (cisplatin, ifosfamide, etoposide) or RT (palliative)	Palliative course
[13]	4 YOF	Paraspinal	L2 paraspinal extradural mass	CT/MRI: L5 soft tissue mass adjacent to bifid lamina and L2 lipoma/epidermoid cyst IVP/US: normal kidneys	L5 Bifid lamina	No teratomatous tissue, but within lipoma, primitive metanephric tissue with tubular and glomeruli epithelium, considered Wilm’s tumorlet	GTR + chemotherapy (10 weeks of weekly vincristine and 2 dactinomycin doses)	Disease‐free at 4 years posttreatment
[14]	2.5 YOF	Paraspinal	2 × 2 cm Subcutaneous lumbosacral mass	CT: normal kidneys	None reported (no NTD)	Embryonic nephric components, central solid nodule with mitotic figures	GTR + chemotherapy (dactinomycin, vincristine)	Disease free at 3 years posttreatment
[15]	4 YOF	Spinal	2.5 cm T12–L4 intradural mass	MRI: diastematomyelia in posterior subarachnoid space CT/US/IVP: normal kidneys	Hypertrichosis, T12–L4 spinal dysraphism, T12–S2 laminar fusion	Fibrous capsule, tubular/glomerular cells, primitive striated muscle fibers in stroma with hyperchromatic nuclei, no nervous or teratomatous components	GTR + chemotherapy (adenine arabinoside‐C, vincristine) + RT	Cerebellar metastasis at 1 year, disease free after 20 months posttreatment for the cerebellar tumor
[16]	2 YOF	Spinal	3‐cm solid L1 intra‐arachnoid mass between bone spur and conus medullaris	Low‐lumbar metrizamide myelography/metrizamide chemotherapy myelography: Intra‐arachnoid cephalic L3 block, tethered cord, T12‐L3 clefting of cord, L1‐L2 clefting of meninges CT/IVP: normal kidneys	Diastematomyelia, hypertrichosis, T12‐L2 intersegmental laminar fusion, spina bifida, bifid L1 spinous process	Undifferentiated tumor lobules enclosing tubular/glomerular components, no nervous tissue	GTR + chemotherapy + RT	Disease free at 1‐year post‐operation
[17]	11YOF	Paraspinal	10 × 12 cm soft tissue sacral lipoma with 6 × 8 cm firm, movable mass at lumbosacral junction	X‐ray: homogenous mass over L4‐S1 spina bifida. Gas myelography: low conus medullaris, L5–S1 focal meningocele. Chest tomogram: metastasis (small peripheral nodules)	L4–S1 spina bifida, 6 × 5 cm spongy sacral mass, lumbar scoliosis, 0.5 cm leg length difference	Vascular tumor on presacral fascia with teratocarcinoma characteristics	GTR + chemotherapy (dactinomycin, Cytoxan, vincristine, adriamycin)	Lung metastasis resolved with chemotherapy and patient did well 10 days postop
[2]	3YOF	Paraspinal	4 × 5 cm Sacrococcygeal region	Radiographs: soft tissue mass from sacrum to left sacrococcygeal area posteriorly	Sacrococcygeal teratoma	Wilms tumor (tubules with epithelial cells) within sacrococcygeal teratoma	GTR + RT (5400 rad over 6 weeks) + chemotherapy (vincristine [0.9 mg/week for 12 weeks], dactinomycin [200 mc/day × 5 every 12 weeks])	Disease free at 46 months after diagnosis

Abbreviations: CT, computed tomography; DOF, day old female; GTR, gross total resection; IVP, intravenous pyelogram; MOF, month old female; MRI, magnetic resonance imaging; NTD, neural tube defect; RT, radiotherapy; STR, subtotal resection; US, ultrasound; WT‐1, Wilm′s Tumor 1 gene; YOF, year old female; YOM, year old male.

Of the reported cases, 81% (13/16) underwent GTR, 81% (13/16) received chemotherapy, and 44% (7/16) received adjuvant radiotherapy (RT). One patient underwent subtotal resection (STR), and one followed a palliative course when there was no response to chemotherapy or RT due to large extension of the tumor. Of the 8 patients receiving chemotherapy with a specified regimen, 4 received vincristine and dactinomycin with no additional chemotherapeutic agent. The following medications were used in the remaining 4 patients: adenine arbinoside‐C (1/4), adriamycin (1/4), carboplatin (1/4), cisplatin (1/4), cyclophosphamide (2/4), doxorubicin (1/4), etoposide (2/4), and ifosphamide (2/4) (Table 2).

While all patients had preoperative imaging (MRI > CT > CXR), the diagnosis of ERWT was rendered only following comprehensive pathologic evaluation (Table 2).

The patients had differing locations of the mass, ranging from T9 through the coccyx. When the spinal level was specified, the most common involved levels were L4 and L5, with a few additional articles indicating the “lumbosacral” area, overall suggesting the greatest prevalence in the lumbosacral region. Moreover, all but 2 patients (88%) had an associated congenital anomaly (Table 2).

## 4. Discussion

ERWT diagnostic criteria was first established in 1978 requiring (1) a triphasic (mesenchymal, epithelial, and blastemal) WT outside the kidney, (2) no teratoid/anaplastic components, and (3) normal kidneys on imaging [18]. There are no diagnostic radiological features for ERWT although US and follow‐up CT/MRI, as appropriate, is a typical imaging paradigm to exclude intrarenal tumors. This makes pathological examination imperative for accurate diagnosis, and this diagnosis is most often made postoperatively, given the above challenges [3, 4].

There are many theories regarding the pathogenesis of ERWT. The “embryonic rest hypothesis of cancer development” was first established by Rudolf Virchow and Julius Cohnheim, suggesting that embryonic cell remnants remaining in organs postembryogenesis can later develop into cancer [7, 19, 20]. Following this hypothesis, ERWT is believed to arise from pluripotent mesenchymal cells, or potentially ectopic nephrogenic rest cells, which develop into nephroblastoma or other malignant masses because of one or more underlying genetic mutations. Indeed, 25% of ERWTs have an identifiable WT1 gene mutation, supporting the notion that ERWT develops from mutated nephrogenic rests [3, 4].

Another proposed mechanism is tumors arising from a teratoma. Teratomas contain a variety of mature and immature tissue elements, and in this hypothesis, immature elements of the teratoma (which may include pluripotent stem cells) could lead to nephroblastoma following additional genetic mutations. While akin to the “embryonic rest hypothesis,” in this proposal, the precursor cells would be present due to the separate neoplastic process, as opposed to residual and/or ectopic embryonic cells. In the present case, there was no evidence of a teratoma following comprehensive review of the submitted mass. Therefore, the most likely explanation is that nephrogenic remnants were present in the paraspinal tissue, suggesting that unknown stimulation could potentially trigger ERWT development from these mesenchymal cells [4].

Paraspinal ERWT in pediatrics are exceptionally rare entities. Due to this, there is no single standardized treatment, and rather a multimodal, case‐specific approach is necessary for ERWT treatment. The National Wilms Tumor Study (NWTS), Children’s Oncology Group (COG) and American Pediatric Surgical Association (APSA) Cancer Committee, initially developed staging and treatment protocols for conventional intrarenal WT. By NWTS, COG, and APSA criteria, Stage I WTs are tumors that are localized to the kidneys; however, ERWTs are found beyond kidney borders and are generally considered Stage II WT. Stage III WT has a positive margin (compared with Stage II which has a negative margin) and is treated with GTR and, despite often favorable histology, chemotherapy (vincristine, dactinomycin, and doxorubicin) and RT for unresectable tumors, recurrent disease, and/or metastasis [4, 21, 22].

Although the above staging system is thought to be applicable to ERWT, accurate staging of ERWT is ultimately unclear due to the extrarenal location and often the lack of an intrarenal tumor. In this case, we found it challenging to appropriately stage our patient. She did not have any detrimental genetic changes commonly seen in WT (such as chromosome 1q gain or 1p/16q LOH which can double the risk of relapse and decrease overall and event free survival) [23–27]. Moreover, although the tumor closely approached a surgical margin as described above, GTR was still achieved with benign final margins. Therefore, because of her age, favorable genetics, and overall histologic findings, we elected to treat this patient like a Stage 1 WT. The patient is periodically being monitored for tumor recurrence, which will inform the need for adjuvant chemotherapy/RT treatment.

## 5. Conclusion

Here, we presented the case of a 0DOF born with an ERWT, tethered spinal cord, and spinal lipoma. She is the 16^th^ case reported for paraspinal/spinal ERWT, 11^th^ case for only paraspinal ERWT, and 4^th^ case diagnosed at ≤ 1 month of age. This is also potentially the first case of reported in utero identification, as this was not a feature of any of the reviewed cases. This case sheds light on the novelty of ERWT, particularly as it requires a pathologic diagnosis rather than an imaging/clinical one. This case highlights (1) the importance of keeping ERWT as a differential diagnostic consideration for spinal/paraspinal tumors and (2) the necessity of establishing a more standardized treatment modality.

## Funding

Authors do not have any financial disclosures and did not receive any funding for completion of this case report.

## Ethics Statement

All work in this manuscript was conducted in accordance with the Declaration of Helsinki (1964).

## Consent

Written consent was obtained from both patients in this case report in accordance with the International Committee of Medical Journal Editors (ICMJE). This case report was exempt from IRB review.

## Conflicts of Interest

The authors declare no conflicts of interest.

## Data Availability

Data sharing is not applicable as no new data were generated.
